# Breeding jassid-resistant okra (*Abelmoschus esculentus*): from morpho-biochemical traits to precision breeding

**DOI:** 10.3389/fpls.2026.1897976

**Published:** 2026-08-13

**Authors:** Fabrice Vihou, Barthélémy B. Yarou, Ya-ping Lin, Xingbo Wu, Mathieu A. T. Ayenan

**Affiliations:** 1 World Vegetable Center, West and Central Africa, Cotonou, Benin; 2 World Vegetable Center, Shanshua, Tainan, Taiwan; 3 Tropical Research and Education Center, University of Florida, Homestead, FL, United States

**Keywords:** *Amrasca* spp., host plant resistance, omic breeding, plant defense mechanisms, trichome density

## Abstract

Okra, an economically important vegetable in tropical and subtropical regions, faces severe yield constraints due to sap-sucking pests, particularly jassids (*Amrasca* spp.). These insects cause hopper burn, leaf curling, and chlorosis, leading to estimated yield losses of 30-100% under severe infestation. Farmers’ heavy reliance on chemical control to manage these insects poses environmental, economic, and health risks, underscoring the need for host-plant resistance as a sustainable component of integrated pest management. Successful breeding for jassid resistance requires in-depth knowledge of its underlying mechanisms. This review synthesizes current knowledge on jassid resistance in okra, focusing on morphological (trichome density, leaf thickness), biochemical (phenolics, defensive enzymes), and physiological traits that confer antixenosis and antibiosis, as well as the largely untapped potential of wild *Abelmoschus* species as reservoirs of novel and durable resistance alleles. We examine advances in genomics, including QTL mapping, genome-wide association studies, and transcriptomics, as tools for dissecting complex resistance mechanisms in okra’s polyploid genome. We further discuss biotechnological and precision breeding strategies such as genomic selection, gene editing, and speed breeding, as avenues to accelerate the development of jassid-resistant cultivars. We conclude by proposing a multi-trait ideotype that integrates morphological, biochemical, and genetic resistance while maintaining high yield and product quality and is supported by an integrated breeding framework that combines conventional and genomic approaches. Key research priorities include discovering new sources of resistance, functionally validating resistance loci, and integrating high-throughput phenotyping with genomic selection to enhance breeding precision and efficiency in okra improvement programs.

## 1 Introduction

Okra (*Abelmoschus esculentus* (L.) Moench) is an economically important vegetable in the Malvaceae family, widely cultivated across tropical, subtropical, and warmer temperate regions. The crop plays a vital role in food security, nutrition, and rural livelihoods, particularly in Africa and Asia, where it serves as both a staple vegetable and an important cash crop (Suma et al., 2023; Singh and Pandey, 2024). Valued for its vitamins, minerals, dietary fiber, and bioactive compounds, okra supports diverse food, pharmaceutical, and processing sectors, especially in Africa and Asia (Mkhabela et al., 2023; Ayenan et al., 2025). Despite its economic and nutritional importance, okra productivity remains constrained by numerous biotic and abiotic stresses, among which insect pests are the most significant yield-limiting factors (Mohapatra et al., 2024; Ounis et al., 2024).

Among the most destructive insect pests are sap-sucking jassids (*Amrasca* spp.), which thrive in hot, dry environments typical of tropical and subtropical okra-growing regions (Nath et al., 2020; Sarsaiya and Singh, 2025). Both nymphs and adults feed on phloem sap, causing chlorosis, leaf curling, hopper burn, reduced photosynthetic activity, flower and fruit abortion, stunted growth, and, under severe infestations, complete crop failure (Nath et al., 2020; Ounis et al., 2024). Yield losses ranging from 30% to 100% have been reported under severe infestations, prompting intensive insecticide use to minimize economic damage (Mohapatra et al., 2024). However, excessive use of chemical control has resulted in insecticide resistance, pest resurgence, adverse effects on beneficial organisms, increased production cost, and risks to human and environmental health (Nath et al., 2020; Ounis et al., 2024). These limitations reinforce the need to strengthen integrated pest management (IPM) through the deployment of durable host-plant resistance.

Host-plant resistance is widely recognized as one of the most effective, economical, and environmentally sustainable approaches for managing insect pests. There is substantial genetic diversity within cultivated okra germplasm and wild relatives, providing a valuable reservoir for breeding resistant varieties without compromising yield or quality (Mkhabela et al., 2023; Suma et al., 2023). Recent advances in genetics and genomics have expanded opportunities to improve insect resistance through quantitative trait locus (QTL) mapping, genome-wide association studies (GWAS), transcriptomics, molecular markers, and modern breeding technologies (Suma et al., 2023; Singh and Pandey, 2024). Despite this progress, research on jassid resistance remains dominated by field-based screening, while the genetic mechanisms, resistance pathways, and breeding strategies required to develop durable resistant cultivars remain fragmented. Realizing this potential requires an in-depth understanding of the mechanisms underlying jassid resistance, together with an integrated framework that combines multiple resistance traits through both conventional and modern breeding technologies, a synthesis that this review aims to provide.

This review is timely because two major developments are reshaping okra breeding and production. First, climate change is contributing to the expansion of jassid distribution, increasing pest pressure and the frequency of outbreaks in many production regions (Shafique et al., 2025). Second, rapid advances in genomics and precision breeding now provide unprecedented opportunities to accelerate the identification, characterization, and deployment of resistance genes in crop improvement programs. Integrating these advances into okra breeding could substantially enhance the efficiency and durability of host-plant resistance.

Accordingly, this review synthesizes current knowledge on the biology, distribution, and impact of jassids in okra; summarizes the morphological, anatomical, biochemical, genetic, and genomic mechanisms underlying resistance; evaluates the contribution of wild Abelmoschus species as sources of novel resistance alleles; and discusses the application of modern breeding approaches, including marker-assisted selection, genomic selection, genome editing, and speed breeding, for developing resistant cultivars. We hypothesize that durable and broad-spectrum resistance to jassids in okra can be achieved by integrating complementary morphological, biochemical, and physiological defense mechanisms with resistance alleles from cultivated and wild *Abelmoschus* germplasm, while leveraging modern genomic and precision breeding technologies. Such an integrated approach is expected to accelerate the development of climate-resilient, high-yielding, and jassid-resistant okra cultivars that support sustainable integrated pest management.

## 2 Methodology

This review was structured around the following research questions: (1) What is the current impact of jassid on okra production systems, and how is the threat of these pests spreading across different regions and cropping systems? (2) Which morphological, anatomical, and biochemical traits underpin antixenosis and antibiosis mechanisms in okra against jassid, and how do these traits contribute to host plant resistance? (3) What is the genetic and genomic basis of jassid resistance in okra, and what role do wild Abelmoschus species play as sources of resistance alleles? and (4) How can modern breeding strategies, including marker-assisted selection, genomic selection, CRISPR/Cas9 gene editing, and speed breeding, be effectively integrated to develop jassid-resistant okra ideotypes compatible with sustainable integrated pest management (IPM) systems? A comprehensive literature search was conducted using Google Scholar and PubMed. The following keywords and their combinations were used: “jassid incidence on okra”, “morphological defense”, “biochemical defense”, “genetic basis of resistance”, “genomic approach for resistance breeding”, and “biotechnology tools effectiveness for breeding”. In total, 200 scientific documents were initially retrieved and downloaded. Titles and abstracts were screened to exclude articles published in non-reputable or predatory journals, as well as papers not directly related to okra-jassid interactions or plant resistance. After full-text assessment, 120 documents that provided relevant and reliable information to address at least one of the four research questions were retained and used to develop this review. Complementary data on the first reports and geographic distribution of jassid incidence on okra worldwide were obtained from the Centre for Agriculture and Biosciences International (CABI) platform. These data were used to contextualize the spatial spread and severity of jassid infestations.

## 3 Global distribution, symptoms, and incidence of jassid in okra

**Figure 1:**
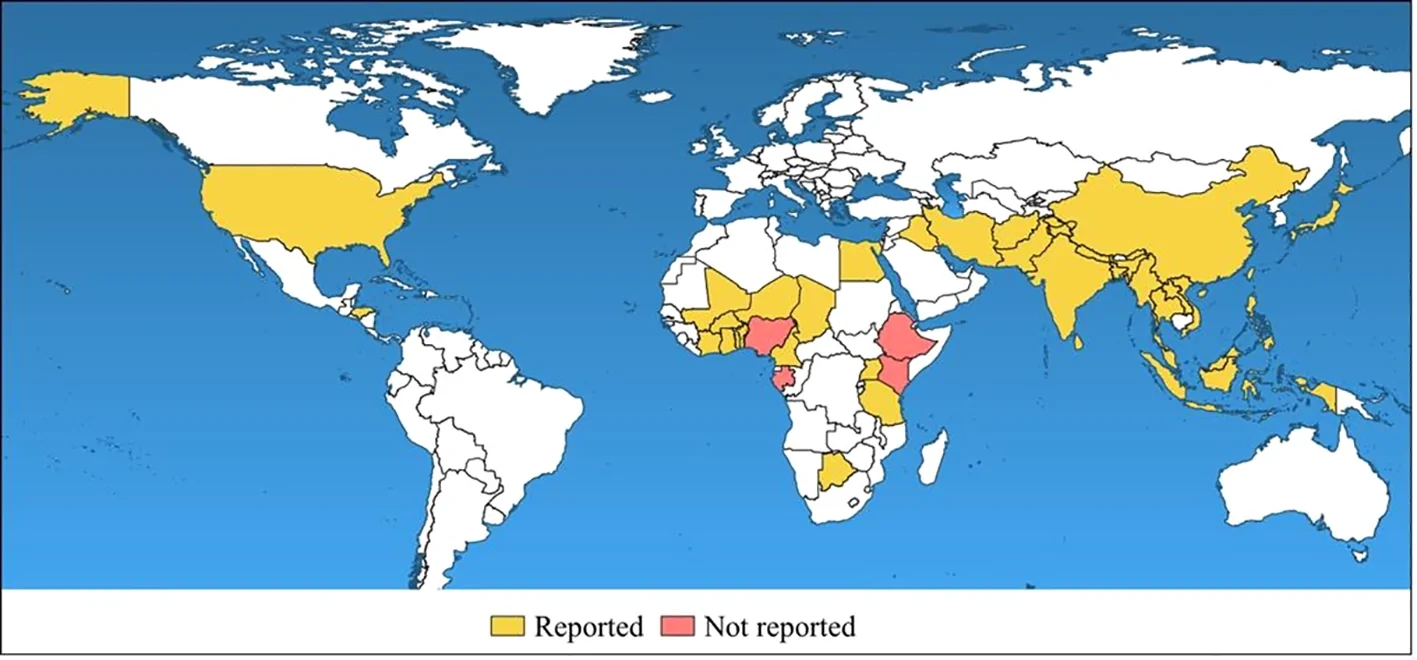
Map of distribution of jassids on okra worldwide (CABI, 2026).

The cotton jassid, *Amrasca biguttula* (Ishida), is a highly polyphagous pest with a vast geographical range spanning Asia, the Middle East, Africa, and South America (**Figure 1**). It poses a critical threat to okra production in both endemic and recently invaded territories (Azrag et al., 2025). Notably, the pest’s actual distribution exceeds the current CABI records; field observations and recent literature confirm its presence in multiple regions currently categorized as ‘Not reported’ (**Figure 1**).

Recent invasion modelling indicates that large cotton and okra-growing belts in Asia, sub-Saharan Africa, and South America are already suitable, or are likely to become suitable for establishment and further spread under future climate scenarios (Azrag et al., 2025). While jassid outbreaks in okra have been documented across South Asia for decades, *A. biguttula* has recently emerged as a devastating invasive species in West and Central Africa, and North America. Specifically in Niger and neighboring countries, these infestations now cause yield losses ranging from 30% to 100%, depending on location and season (Akonde et al., 2024). Since early 2025, *A. biguttula* has landed south Florida in the United States through the Caribbean and has caused significant damages to a range of important crops in the Malvaceae family including okra, tropical hibiscus, and cotton (Liburd et al., 2024; Attia and Joseph, 2025; Esquivel et al., 2025).

**Figure 2:**
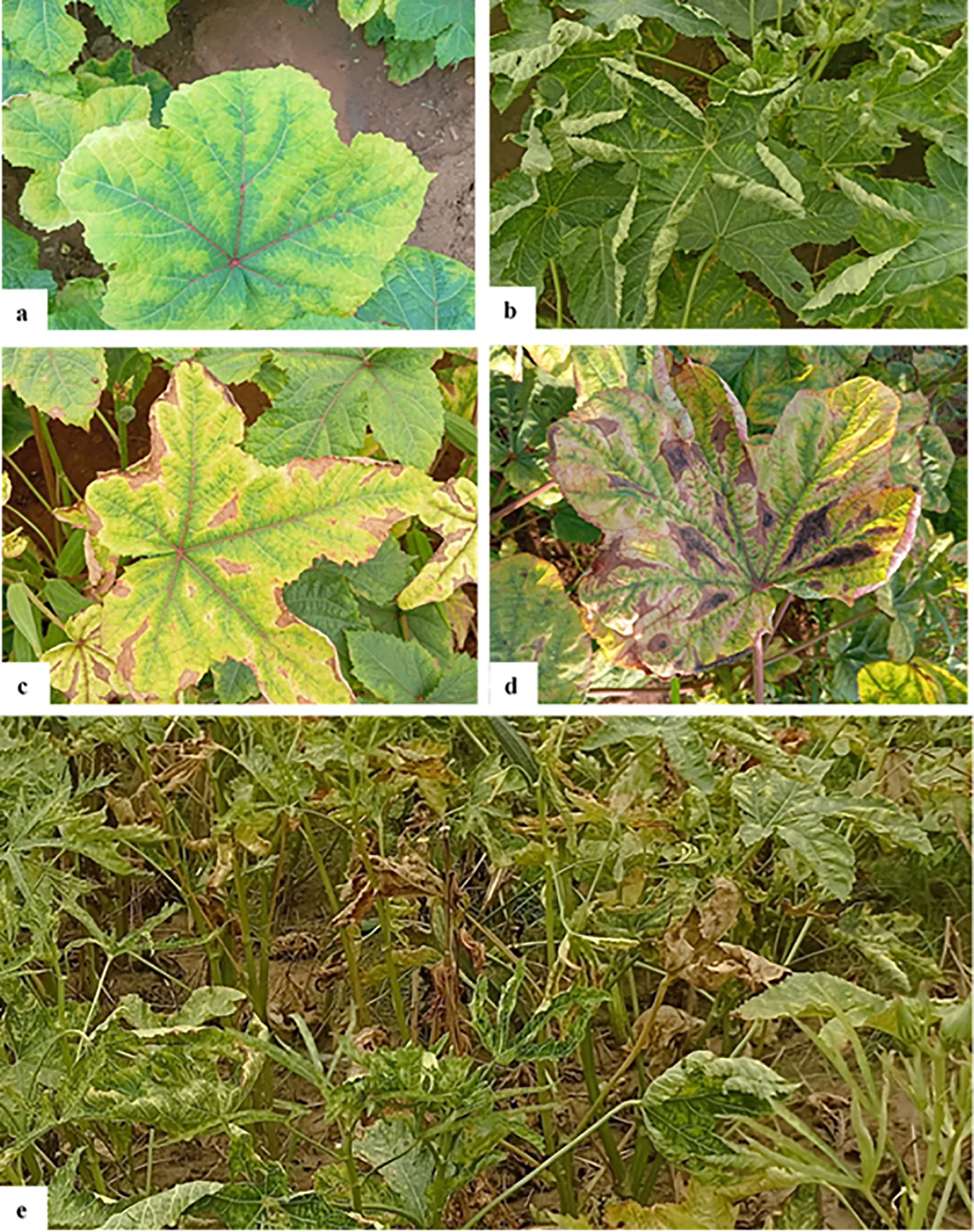
Symptoms of jassids’ attack on okra: **(a)** marginal yellowing and reddening **(b)** leaf curling and wrinkling; **(c)** tip burn; **(d)** stunting, drying of leaves, and **(e)** complete desiccation of plants and abortion of flowers (photo credit: Fabrice Vihou).

**Figure 3:**
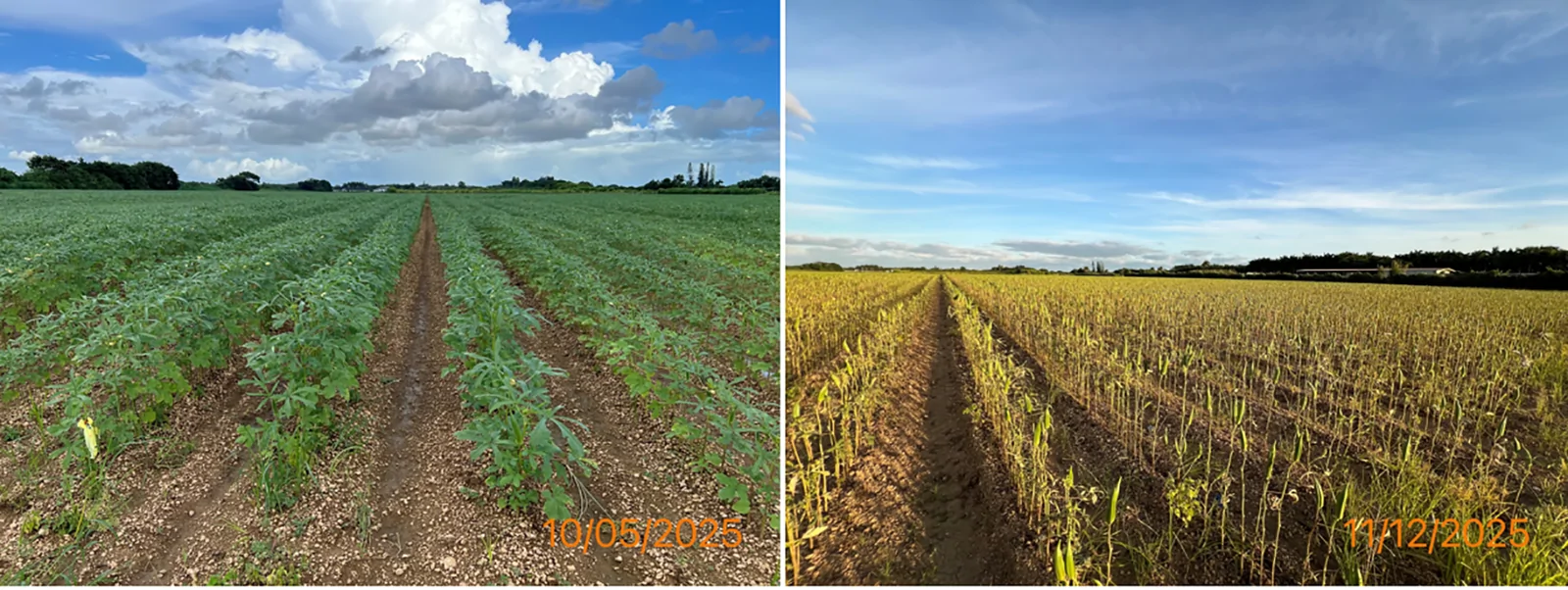
Integrated breeding pipeline for resistance to jassids.

Both nymphs and adults feed on the undersides of leaves and inject salivary phytotoxins, resulting in a characteristic complex of symptoms on okra that includes marginal yellowing and reddening (**Figure 2a**), leaf curling and wrinkling (**Figure 2b**), tip burn (**Figure 2c**), stunting, drying of leaves (**Figure 2d**) and, in severe cases, complete desiccation of plants and abortion of flowers and young fruits (**Figure 2e**) (Singh et al., 2018; Akonde et al., 2024). Population dynamics studies on okra prove that jassid incidence typically begins within two weeks after crop emergence and can persist until harvest, with peak densities often observed during warm, relatively dry periods; populations are positively associated with high maximum temperatures and vapor pressure deficit, while high relative humidity and rainfall tend to suppress abundance (Saroj et al., 2017). In heavily infested fields, jassid densities often exceed the economic threshold of two nymphs per leaf (Nyesse et al., 2026), and uncontrolled infestations can cause substantial yield losses (**Figure 3**). Consequently, jassids are considered one of the most important sucking pests constraining okra productivity across tropical and subtropical production systems worldwide (Akonde et al., 2024; Azrag et al., 2025).

## 4 Morphological defense traits conferring jassid resistance

### 4.1 Trichomes, leaf surface traits, leaf texture, thickness, and coloration

**Figure 4:**
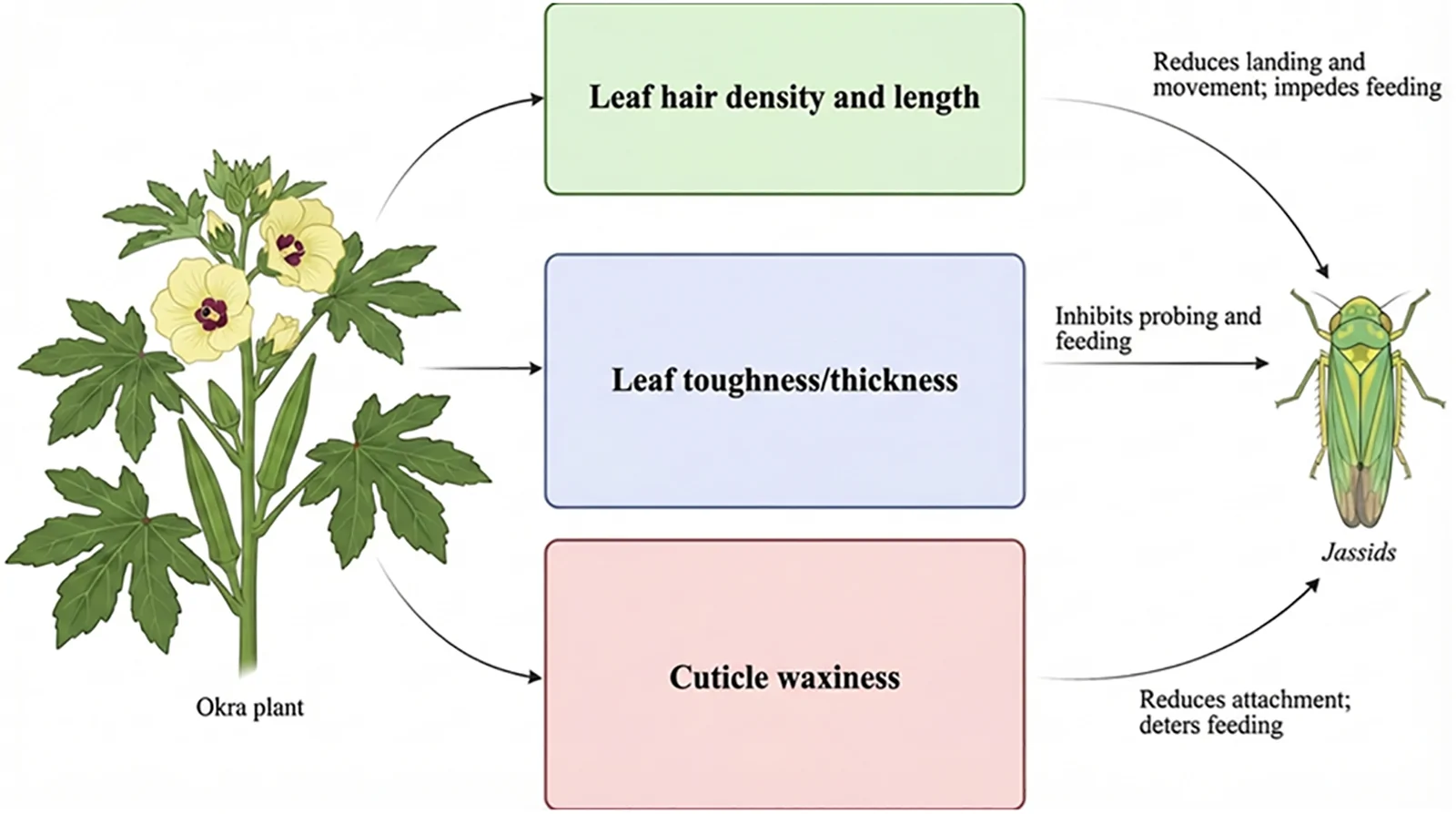
Schematic representation of key okra morphological traits and their effects on jassids.

Trichomes and associated leaf surface traits are a central component of morphological defense in okra against jassid, operating through both antixenosis and antibiosis (**Figure 4**). Comparative evaluations across diverse okra genotypes consistently demonstrate a strong negative relationship between jassid incidence and trichome density on the lamina, midrib, and veins, with tolerant or resistant lines bearing significantly higher numbers of hairs per unit area than susceptible cultivars (Halder et al., 2016; Barman et al., 2024). Beyond density, trichome architecture is critical as resistant genotypes typically express longer, stiffer, and more erect non-glandular trichomes, and both trichome length and density show significant negative correlations with jassid population density, indicating reduced landing, mobility, probing efficiency, and oviposition (Sandhi et al., 2017; Yogesh and Padhi, 2022). Similar associations between enhanced trichome and reduced jassid damage have been widely reported in upland cotton and other Malvaceae, reinforcing the general importance of hairiness as a conserved resistance trait against sap-feeding leafhoppers (Kumbhalkar et al., 2022; Subhashini et al., 2025). Functionally, dense trichomes act as physical barriers, restricting access to preferred feeding and oviposition sites such as veins and midribs, thereby conferring strong non-preference. Negative correlations between trichome density on these tissues and jassid abundance provide direct evidence for antixenosis as a dominant mechanism (Halder et al., 2016; Sandhi et al., 2017; Acharya et al., 2019). Trichome-mediated resistance is further reinforced by biochemical interactions: trichome-led tolerant genotypes frequently exhibit elevated phenolic content, and jassids that establish on such plants show reduced survival, development, and population build-up, indicating a complementary antibiosis effect (Sandhi et al., 2017; Acharya et al., 2019; Barman et al., 2024; Prithiva et al., 2024).

**Figure 5:**
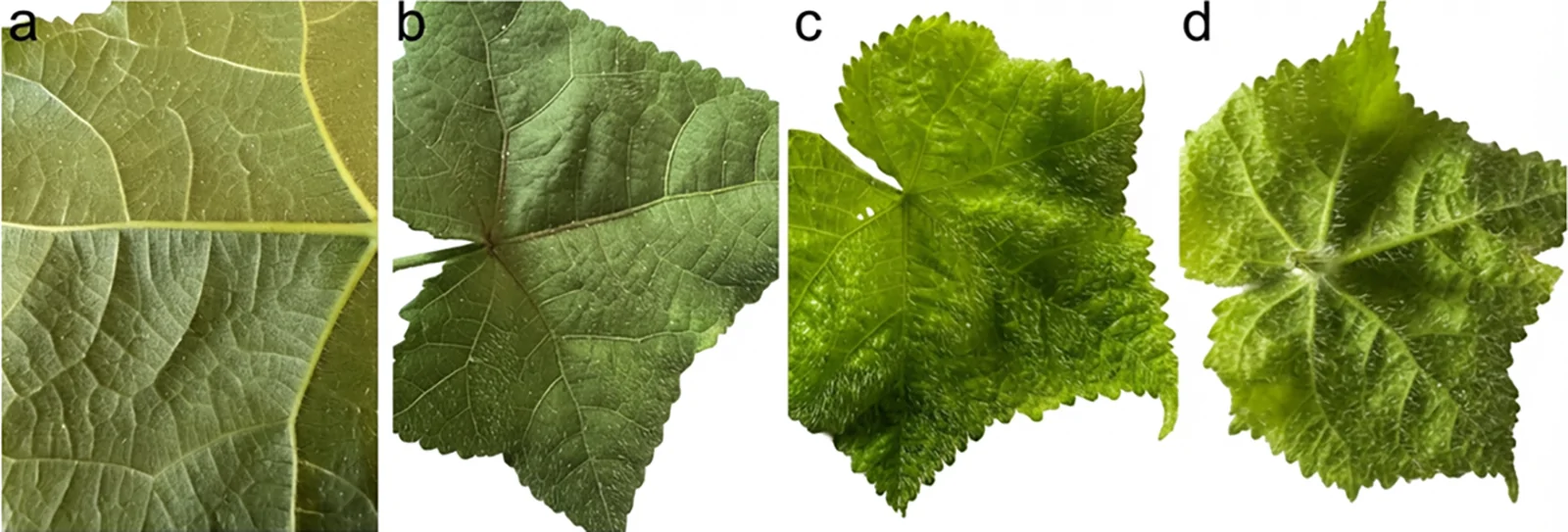
Leaf trichomes **(a)** no hairs, **(b)** medium hairs **(c, d)** profuse hairs (photo credit: Fabrice Vihou).

In addition to trichomes, leaf thickness, texture, and surface properties contribute significantly to jassid tolerance by shaping both host preference and insect performance (**Figure 5**). Leaf lamina thickness is generally negatively correlated with jassid infestation, likely because tougher tissues impede stylet penetration and reduce sap extraction efficiency (Acharya et al., 2019; Yogesh and Padhi, 2022). However, this effect is tissue-specific: increased midrib thickness may favor jassid incidence by providing larger tissue volume and structurally stable oviposition sites, thereby enhancing egg deposition and nymphal establishment (Halder et al., 2016). This contrast highlights that tolerance depends not only on overall toughness but also on its spatial distribution across the leaf. Leaf surface properties are further modulated by the quantity and organization of epicuticular waxes, which form the first interface between the insect and the plant. Across crops, increased wax deposition modifies surface roughness, wettability, and mechanical strength, often impairing insect walking, attachment, probing behavior, and host recognition by piercing-sucking pests (Dhanyalakshmi et al., 2019). Studies in sorghum and maize demonstrate that changes in wax chemistry and structure can markedly alter aphid and leafhopper settling and feeding patterns, while also revealing trade-offs between physical barriers and induced chemical defenses (Liu et al., 2024). By analogy, okra genotypes with thicker lamina, robust wax layers, reduced glossiness, and optimized trichome architecture are likely to express strong antixenosis through reduced attractiveness and impaired colonization, while simultaneously imposing antibiosis via reduced feeding efficiency and nymphal performance (Halder et al., 2016; Acharya et al., 2019; Barman et al., 2024).

Overall, breeding for durable jassid tolerance in okra is best achieved by pyramiding favorable leaf surface traits, including high trichome density and length on lamina, veins, and midribs, controlled midrib thickness, increased lamina toughness, and robust cuticular wax deposition. Integrating these complementary morphological attributes as explicit selection criteria in okra breeding programs offers a biologically grounded and sustainable pathway to enhance resistance through combined non-preference and reduced pest fitness.

### 4.2 Plant architecture and phenology

Okra plant architecture and phenology also contribute to morphological defense against cotton jassid by modifying host location, colonization, and the temporal window of exposure. Biophysical assessment of okra germplasm indicates that plant height, number of leaves, and total leaf area are positively associated with jassid incidence, as taller, more vigorously growing plants with larger canopies provide greater feeding and oviposition surface and support higher nymphal populations (Halder et al., 2016; Yogesh and Padhi, 2022). In contrast, more compact genotypes with fewer leaves and reduced canopy area tend to harbor lower jassid densities, suggesting that a relatively open, less luxuriant canopy can limit colonization pressure and within-plant population build-up (Halder et al., 2016). Studies on physico-morphic resistance further prove that tolerant cultivars such as Arka Anamika (*Abelmochus esculentus*, cultivated varieties in India) maintain lower jassid populations despite comparable agro-climatic conditions, implying that their particular combination of growth habit and leafiness, together with defensive surface traits, contributes to antixenosis by making plants less suitable or less apparent to immigrating adults (Acharya et al., 2019).

Phenological patterns strongly mediate escape mechanisms and reduced exposure. Population dynamics studies in South Asia show that jassid pressure peaks during specific weeks of the kharif season, and that infestation is closely tied to the vegetative to early reproductive stages when young foliage is abundant (Sharma et al., 2018; Deepak and Patel, 2024). Genotypes or cropping schedules that reach key susceptible stages either earlier or later than the regional jassid peak can therefore partially escape damaging densities, as seen where seasonal shifts markedly alter sucking-pest loads on the same cultivars (Deepak and Patel, 2024). Early flowering and pod set, combined with moderate plant stature and less dense foliage, thus represent useful ideotypes that shorten the overlap between maximum jassid abundance and the most vulnerable crop stages, reducing cumulative feeding time and injury (Acharya et al., 2019; Barman et al., 2024). The jassid population dynamics are currently poorly understood in Africa. A better understanding of jassid population dynamics and integrating selection for architectural and phenological traits, along with leaf-level resistance factors, could strengthen host plant resistance and provide a durable, low-input component of integrated jassid management in okra.

## 5 Biochemical and physiological defense mechanisms

### 5.1 Secondary metabolites and allelochemicals

Biochemical and physiological defense in okra against jassids is strongly mediated by secondary metabolites and allelochemicals, particularly phenolics, flavonoids, tannins, and alkaloids, which act through feeding deterrence and toxicity (Barman et al., 2024). Polyphenols (including phenolic acids, flavonoids, and tannins) are among the most widespread defensive compounds, accumulating constitutively or being induced after herbivore attack, where they reduce tissue palatability, interfere with digestion, and directly impair insect growth and survival (Upadhyay et al., 2025). Both phenylpropanoids and flavonoids can act as phytoanticipins (pre-formed barriers) and phytoalexins (inducible toxins), with their efficacy depending on localization in epidermal and vascular tissues, sustained availability, and synergistic interactions among individual molecules (Kumar et al., 2023). Elevated phenolic and flavonoid contents have been repeatedly associated with resistance to sap-sucking insects such as aphids and thrips, where high levels decrease probing, shorten feeding bouts, and increase mortality or developmental delays (Farhan et al., 2024; Sunidhi et al., 2025). Tannins, as high-molecular-weight phenolics, exert strong anti feedant effects by binding dietary and salivary proteins, lowering nutrient availability, and causing gut damage, while their bitter taste further discourages sustained feeding (Khan et al., 2025). Alkaloids, a major nitrogen-containing class, typically act at low concentrations as neurotoxins or enzyme inhibitors, disrupting neurotransmission, hormonal regulation, and redox balance in insects, with broad insecticidal and repellent activity documented from multiple crop and wild species (War et al., 2020). Overall, these metabolites contribute to antixenosis by deterring colonization and reducing stylet insertion, and to antibiosis by impairing survival, fecundity, and population build-up of piercing-sucking pests, including jassid. Although specific profiles in okra-jassid systems remain underexplored, evidence from related plant-hemipteran interactions indicates that breeding for high and inducible pools of phenolics, flavonoids, tannins, and selected alkaloids, supported by metabolomic screening, offers a robust avenue to reinforce biochemical resistance and reduce reliance on synthetic insecticides in okra-based production systems (Jahan et al., 2025).

### 5.2 Defensive enzymes and oxidative responses

Biochemical and physiological defense of okra against jassids involves a tightly regulated oxidative burst and activation of key antioxidant enzymes, including peroxidase (POD), polyphenol oxidase (PPO), and catalase (CAT). Upon piercing-sucking injury, plants rapidly generate reactive oxygen species (ROS), mainly superoxide and hydrogen peroxide, which act as early signals to activate downstream defense pathways and reprogram primary metabolism, including photosynthesis, in favor of defense rather than growth (Haghpanah et al., 2025). Localized ROS accumulation around feeding sites is a hallmark of herbivore recognition and can restrict pest performance directly through oxidative damage or indirectly by driving the synthesis and oxidative activation of phenolic defense metabolites (Sperdouli et al., 2022). In this context, POD contributes to hydrogen peroxide detoxification, cross-linking of cell-wall phenolics and lignification, thereby strengthening physical barriers, while PPO promotes the oxidation of phenolics to reactive quinones that reduce nutritive value and cause oxidative stress in the insect gut (Zhang and Sun, 2021).

At the same time, excessive ROS must be controlled to avoid self-toxicity, and okra, like other crops, relies on a coordinated antioxidant enzyme network in which CAT, POD, and other peroxidases scavenge surplus hydrogen peroxide and maintain redox homeostasis (Dvořák et al., 2021). Fine-tuning of this balance is achieved through ROS-dependent signaling cascades that interact with phytohormones such as jasmonic and salicylic acids, as well as with calcium and MAPK signaling, integrating insect-derived cues into systemic resistance responses (Yash et al., 2025). Evidence from sap-sucking insect systems shows that a rapid but transient ROS burst, followed by sustained up-regulation of antioxidant enzymes, underlies enhanced tolerance and photoprotective adjustments in photosystem II, limiting oxidative damage while preserving signaling capacity (Sperdouli et al., 2022). Extrapolating to okra-jassid interactions, genotypes capable of mounting strong POD/PPO responses coupled with efficient CAT-mediated detoxification and precise ROS-mediated signaling are likely to exhibit superior biochemical resistance and should be prioritized in breeding programs targeting durable jassid management.

### 5.3 Nutritional quality and insect performance

Biochemical and physiological defense of okra against jassid is closely linked to the nutritional quality of leaf tissues, particularly nitrogen, sugars, and amino acid-derived compounds, which collectively shape jassid fitness and underpin trade-offs between resistance and productivity (Barman et al., 2024). Field and laboratory studies revealed that higher leaf protein and nitrogen contents were positively associated with jassid abundance, indicating that nitrogen-rich foliage provides superior resources for nymphal development and adult fecundity (Iqbal et al., 2011; Barman et al., 2024). In a detailed chemical analysis of leaf of nine okra genotypes, crude protein and nitrogen showed strong positive correlations with jassid population, while fiber fractions and structural carbohydrates were negatively related, suggesting that dilution of readily assimilable nitrogen within less digestible cell-wall components can reduce pest performance (Iqbal et al., 2011). Similarly, comparative screening of okra hybrids revealed a positive correlation of jassid and whitefly population with total protein and total sugar content, whereas more resistant lines combined lower soluble nutrients with higher levels of phenolics and greater hair density, supporting an integrated role of nutritive and defensive traits in antixenosis and antibiosis (Barman et al., 2024). Screening of wild and cultivated *Abelmoschus* spp. further indicates that total sugars and their partitioning into reducing versus non-reducing forms influence susceptibility. In fact, jassid resistance was associated with high total sugars but low reducing sugar levels, together with elevated phenols, tannins, and silica, implying that carbohydrate form and its interaction with secondary metabolites modulate phloem palatability and insect fitness (Sandhi et al., 2017).

Beyond constitutive leaf chemistry, external nitrogen supply can strongly modify these nutritional landscapes and jassid pressure. On-farm experiments in Nepal demonstrated that increasing nitrogen fertilization from 100 to 200 kg N ha^−1^ significantly elevated jassid population and damage scores, whereas lower N input combined with late sowing minimized pest build-up (Regmi et al., 2020). Integrated nutrient management studies similarly report that regimes designed primarily to maximize yield through high mineral N or nitrogen-rich organic inputs often coincide with higher populations of sucking pests, whereas balanced combinations of inorganic fertilizers with neem-based amendments can sustain yield while suppressing jassid and other pests (Mishra et al., 2020). These findings overall highlight a trade-off between resistance and productivity. High nitrogen and soluble sugar status enhance vegetative growth and fruit yield but simultaneously improve host quality for jassids, whereas genotypes and management strategies that moderate leaf nitrogen and adjust sugar profiles, in concert with elevated structural and secondary compounds, tend to constrain pest fitness at some potential cost to maximum yield (Iqbal et al., 2011; Sandhi et al., 2017; Barman et al., 2024). For sustainable jassid management in okra, breeding and fertilizer regimes therefore need to target an optimized intermediate nutritional status that supports acceptable productivity while limiting phloem nutritional quality for the pest.

## 6 Genetic basis of jassid resistance in okra

### 6.1 Inheritance patterns of jassid resistance in okra

The genetic architecture of jassid tolerance in okra reflects a combination of additive and non-additive gene effects, shaping both breeding strategy and the stability of tolerance across environments. Early generation-mean analyses demonstrated that tolerance to cotton jassid is predominantly governed by dominant genes, with significant additive and dominance components. Notably, additive effects and dominance × dominance epistasis accounted for the largest share of phenotypic variation, highlighting opportunities for both direct selection and the exploitation of heterosis (Sharma and Gill, 1984). Subsequent studies in okra and cotton corroborate this finding, indicating that jassid resistance is frequently controlled by dominant loci with appreciable additive contributions, often modulated by duplicate or inhibitory epistatic interactions (Sharma and Gill, 1984; Patil et al., 2017; Yaksha et al., 2022). Observed segregation ratios (3:1 or 13:3 resistant: susceptible) further support simple to digenic inheritance patterns in segregating generations (Sharma and Gill, 1984; Patil et al., 2017; Yaksha et al., 2022). Complementary evidence from interspecific and upland cotton populations underscores the prevalence of epistatic and inhibitory interactions among resistance loci, where pubescence- and hairiness-related genes (e.g., H_1_ and associated modifiers) play a central role in heritable jassid resistance (Patil et al., 2017; Subhashini et al., 2025).

Parallel work on resistance to other biotic stresses in okra, such as yellow vein mosaic virus and enation leaf curl disease, reveals duplicate dominant gene systems and substantial dominance × dominance interactions, reinforcing the observation that non-additive gene action is important for defense-related traits and that transgressive segregants can be generated through recombination followed by delayed selection (Maurya et al., 2025; Singh et al., 2025). Genotypic evaluations across seasons and locations for sucking pests reveal significant genotype × environment interactions for pest pressure and yield, showing that relative susceptibility or tolerance can shift with season and site (Shitel et al., 2023; Abang et al., 2024). These interactions underline the need to assess putatively jassid tolerant genotypes across contrasting environments. Overall, the available evidence suggests that jassid tolerance in okra is under complex inheritance with both additive and non-additive components, and that durable tolerance will require simultaneous exploitation of additive variance through line development and non-additive variance through heterosis breeding, tested under multi-environment trials to account for genotype × environment interactions.

### 6.2 Sources of jassid resistance in okra

The genetic basis of jassid resistance in okra is strongly informed by diverse germplasm sources, including landraces, wild relatives, and advanced breeding lines, many of which also provide insights into the stability of resistance across environments. Multi-season and multi-species screenings have shown that cultivated *A. esculentus* is generally susceptible, whereas several wild *Abelmoschus* taxa consistently express moderate to high resistance to jassids and associated sucking pests (**Table 1**). In field evaluations over two to three seasons, accessions of *A. moschatus, A. tetraphyllus, A. manihot, and A. angulosus*, along with specific wild accessions such as IC141055 (**Table 1**), were identified as resistant or tolerant to jassids, often in combination with resistance to whitefly, fruit and shoot borer, and yellow vein mosaic virus, highlighting their value as donors of broad-spectrum and potentially durable resistance (Prabu et al., 2009; Sandhi et al., 2017; Badiger and Yadav, 2019). Germplasm screening further demonstrated that *A. moschatus* and *A. ficulneus* are highly resistant, while interspecific hybrids and amphidiploids involving *A. tetraphyllus* and *A. manihot* spp. *tetraphyllus* can transfer jassid tolerance into cultivated backgrounds, indicating that resistance is introgressible and at least partially stable across generations (Prabu et al., 2009).

**Table 1:** Available sources of resistant genotypes to jassid.

Species/accessions	Origin	Type	Trichomes density	Phenol content	Status	References
*Abelmoschus tetraphyllus*	India	Wild	Dense, long trichomes (4.75-7.50/unit, very pubescent)	High total phenols (≈1.52-1.58 mg g^−1^)	High field resistance (low nymphal population, low injury index)	Sandhi et al. (2017)
*Abelmoschus angulosus*	India	Wild	Dense, long, and erect trichomes	High total phenols	High resistance (1.56–1.99 nymphs/leaf; susceptibility index ≈2.7–2.9)	Sandhi et al. (2017)
*Abelmoschus moschatus*	India, Rahuri	Wild	High pubescence (not quantified)	Not indicated	Resistant (lower nymph population per leaf)	Prabu et al. (2009)
*Abelmoschus moschatus* (IC141055)	IARI, New Delhi (India)	Wild	Not specified (probably high pubescence)	Not indicated	Resistant	Badiger and Yadav (2019)
*Abelmoschus tuberculatus*	India	Wild	Moderately dense trichomes	Moderate total phenols	Moderately resistant in the field	Sandhi et al. (2017)
*Abelmoschus manihot*	India	Wild	Moderate trichomes	Moderate total phenols	Moderately resistant	Sandhi et al. (2017)
*Abelmoschus esculentus* 7 (IC050602)	PAU Ludhiana (India)	Cultivated	Moderate trichomes	Moderate total phenols	Moderate resistance (intermediate population and index)	Sandhi et al. (2017)
*Abelmoschus esculentus* (EC0306728)	PAU Ludhiana (India)	Cultivated	Moderate trichomes	Moderate total phenols	Moderately resistant	Sandhi et al. (2017)
*Abelmoschus esculentus* (VROB-181)	IIVR India,	Cultivated	Higher trichome density than sensitive controls (≈11.9–10 trichomes cm^−2^ on lamina/vein)	Very high total phenols (75.04 mg 100 g^−1^)	Tolerant (5.43 jassids/leaf; resistant category)	Halder et al. (2016)
*Abelmoschus esculentus* (VROT-108)	India, IIVR	Cultivated	Very high trichome density (16.35 cm^−2^)	High total phenols (63.13 mg 100 g^−1^)	Tolerant (low population of Jassids)	Halder et al. (2016)
*Abelmoschus esculentus* (Kajari NOH-1684)	India	Commercial hybrid	Denser and longer trichomes	High total phenols, lower sugars	Relatively resistant variety (low infestation)	Barman et al. (2024)
*Abelmoschus esculentus* (Japani Jhar)	India	Commercial hybrid	Dense trichomes	High phenols	Relatively resistant	Barman et al. (2024)
*Abelmoschus esculentu*(Singham)	India	Commercial hybrid	Dense trichomes	High phenols	Relatively resistant	Barman et al. (2024)
*Abelmoschus esculentus* (Rohini)	India	Commercial hybrid	Dense trichomes	High phenols	Relatively resistant	Barman et al. (2024)
*Abelmoschus esculentus (*BBSR-37)	India	Cultivated	Dense trichomes	High phenols	Resistant	Yogesh and Padhi (2022)
*Abelmoschus esculentus* (BBSR-36)	India	Cultivated	Dense trichomes	High phenols	Resistant	Yogesh and Padhi (2022)
*Abelmoschus esculentus* (BBSR-57)	India	Cultivated	Dense trichomes	High phenols	Resistant	Yogesh and Padhi (2022)
*Abelmoschus esculentus* (Pusa A-4)	India	Cultivated	Dense trichomes	High total phenols, lower sugars	Resistant	Yogesh and Padhi (2022)
*Abelmoschus esculentus* (Arka Anamika)	India	Cultivated	Dense trichomes	High total phenols, lower sugars	Resistant	Acharya et al. (2019)

Within cultivated gene pools, differential varietal responses across locations and seasons indicate substantial genotype × environment interaction but also reveal breeding lines and varieties with relatively stable tolerance. Repeated field screening has consistently identified genotypes such as Arka Anamika, BCO-1, Kajari NOH-1684, Japani Jhar, Singham, Rohini, and institutional lines BBSR-37, BBSR-36, BBSR-57, and Pusa A-4 (**Table 1**) as showing lower jassid incidence or injury indices, with resistance linked to favorable physico-morphic and biochemical traits (Acharya et al., 2019; Yogesh and Padhi, 2022; Barman et al., 2024). Early varietal work also reported selections such as New Selection, Sel-2-2, and Sel-1 as tolerant materials (**Table 1**), maintaining reduced jassid populations and damage under both field and screenhouse conditions, suggesting a degree of environmental stability (Bindra and Mahal, 1979). However, other varietal evaluations across different agro-climatic regions often find all commercial cultivars susceptible or only moderately tolerant, underlining that resistance is quantitative and sensitive to local pest pressure and climate (Sultana et al., 2017). Together, these studies indicate that wild *Abelmoschus* species and a small set of tolerant landraces and breeding lines constitute the primary genetic reservoirs of jassid resistance, and that achieving stable resistance across environments will depend on systematic introgression from wild relatives combined with multi-environment testing of derived lines and hybrids. However, these studies have mostly been limited to Asia, with little knowledge of how okra germplasm responds to jassid in African environments, an emerging hotspot for the pest.

**Figure 6:**
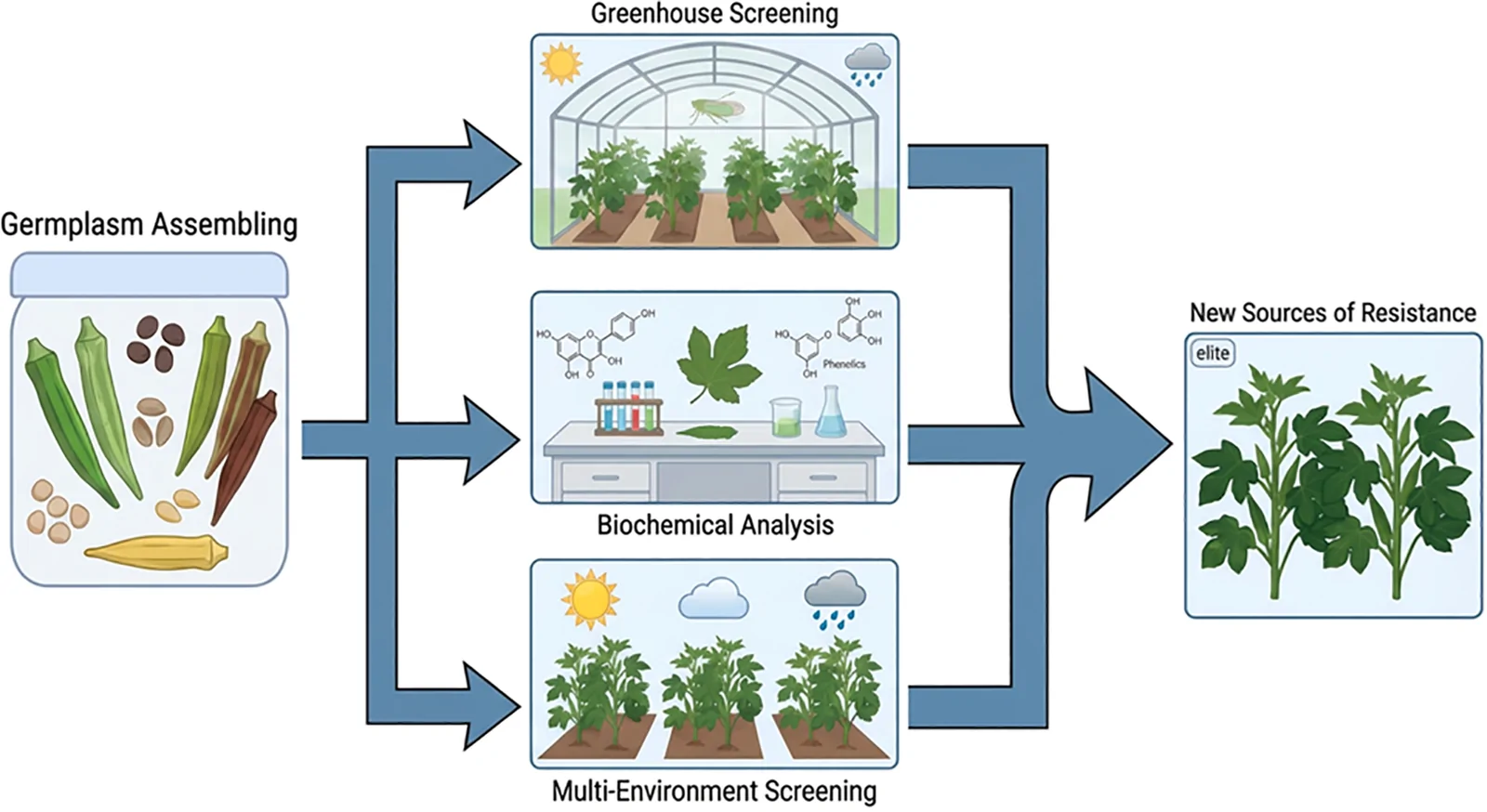
Pathway for the identification of new sources of resistance to jassid.

With the increasing global incidence and spread of jassid infestations, there is an urgent need to identify new sources of resistance to effectively manage these pests and reduce reliance on synthetic pesticides, whose excessive use poses serious risks to human health and the environment. In this context, several complementary approaches can be considered for harnessing resistance in okra. One strategy is the assembly and characterization of diverse germplasm sets encompassing cultivated and wild relatives from major centers of diversity such as Africa, Asia, and America, which can broaden the genetic base for jassid tolerance or resistance (**Figure 6**). Such collections may be evaluated under controlled greenhouse conditions, where artificial jassid inoculation enables precise and uniform assessment of plant responses, and in parallel under field conditions across hotspot environments to capture variation under natural infestations and identify stable resistance (**Figure 6**). These phenotyping efforts can be complemented by biochemical analyses to elucidate resistance-associated traits, including morphological and physiological mechanisms (**Figure 6**).

### 6.3 Breeding trade-offs: managing pleiotropy in jassid-resistant okra

Breeding okra for resistance to jassid involves managing pleiotropic traits whose effects extend beyond pest resistance, often generating trade-offs with yield and fruit quality. Morphological traits such as high trichome density and length on lamina, midrib and veins, and increased leaf thickness consistently show negative correlations with jassid incidence, indicating strong antixenosis and antibiosis, but these traits can be associated with altered canopy architecture and potential yield penalties that may not suit intensive production systems or market preferences (Halder et al., 2016; Yogesh and Padhi, 2022). For example, resistant genotypes or cultivars such as Arka Anamika and several wild *Abelmoschus* species combine high trichome density, erect hairs, broad leaves and elevated phenolics with significantly lower jassid populations, but some of these genotypes also show lower yield potential or less desirable horticultural traits than commercial checks, highlighting a trade-off between resistance and marketable productivity (Sandhi et al., 2017; Acharya et al., 2019; Badiger and Yadav, 2019). Consequently, selecting for compact, small-leaf, thin-midrib ideotypes to reduce jassid attack may inadvertently depress yield potential or harvest index. Biochemically, higher levels of total phenols and tannins are linked to reduced jassid density, but may also influence leaf palatability, pod texture or processing quality, forcing breeders to balance defensive chemistry with culinary and nutritional attributes (Halder et al., 2016; Sandhi et al., 2017; Barman et al., 2024), especially when breeding for region where okra leaves are also consumed as vegetables. In addition, preliminary screening of U.S. okra germplasm revealed that tolerance is associated with photosensitivity, adding further complexity to breeding improved cultivars (personal communication). Evidence of multiple resistance to jassid and other pests (whitefly, fruit and shoot borer, and viruses) in wild accessions and selected lines further underscores pleiotropy. Genotypes such as IC141055 or BCO-1/Arka Anamika provide broad biotic stress resistance but may require substantial trait recovery to match elite yield and pod quality standards (Acharya et al., 2019; Badiger and Yadav, 2019). Contemporary breeding frameworks for okra in Africa explicitly emphasize integrating biotic resistance traits with value-added and market traits, recognizing that resistance-conferring morpho-biochemical traits are often pleiotropic and must be combined carefully to avoid sacrificing farmer and consumer acceptance (Ayenan et al., 2025).

## 7 Genomic approaches to dissect and deploy jassid resistance

### 7.1 QTL mapping and linkage analysis

Genomic approaches offer a promising framework for elucidating the genetic basis of jassid resistance in okra. However, progress remains limited by several inherent challenges. The complex polyploid genome of okra, combined with difficulties in developing appropriate mapping populations and obtaining reliable, high-quality phenotypic data, continues to hinder the genetic dissection of resistance traits. jassid resistance is generally regarded as a quantitative trait controlled by multiple loci with small to moderate effects, which complicates its detection. Therefore, linkage-based QTL mapping in okra requires large, well-structured biparental or multiparent populations and high-density genome-wide SNP genotyping (Kaur et al., 2021). Studies in other crops have demonstrated that dense linkage maps generated through genotyping-by-sequencing (GBS) or SNP arrays can significantly improve QTL detection and mapping resolution for pest and disease resistance traits (Saxena et al., 2017). However, polyploid genomes require specialized statistical frameworks that can estimate allele dosage, multivalent pairing, and complex segregation patterns (Liao et al., 2021). In autotetraploid and higher-ploidy systems, approaches based on random effects, identity-by-descent QTL models, and probabilistic genotype calling have shown promise in improving marker informativeness and QTL detection (Liao et al., 2021; Wijnen et al., 2023). These advances are especially relevant to okra, where the allopolyploid nature of *Abelmoschus esculentus* complicates recombination dynamics, marker phasing, and the estimation of QTL effects.

Simultaneously, phenotyping for jassid resistance remains a major bottleneck in okra research. Studies on okra and related species consistently report significant genotype by environment interactions and highly variable pest pressure, which compromise the reliability and reproducibility of resistance assessments (Barman et al., 2024; Subhashini et al., 2025). Current phenotyping approaches are often inconsistent across studies, limiting the comparability of results and the power to detect stable QTLs. Quantifying plant responses to jassid damage is critical for QTL identification, given the pest’s high mobility and the confounding effects of concurrent abiotic and biotic stresses. To address these limitations, there is a clear need for standardized and robust phenotyping frameworks, including multi-environment trials, standardized scoring systems, controlled or artificial infestation, and repeated measurements analyzed with mixed models to estimate best linear unbiased predictions (BLUPs) (Kaur et al., 2021; Delvento et al., 2023). Integrating polyploid-aware mapping approaches with rigorous physiological phenotyping will be essential for identifying stable QTLs and tightly linked markers for marker-assisted selection (MAS) and genomic selection (GS) toward durable jassid resistance in okra. However, such integration remains limited, constraining progress in marker development and deployment. The effective utilization of these loci in polyploid backgrounds requires dense genome-wide marker coverage and genotyping platforms that account for allele dosage and complex inheritance patterns. Evidence from other polyploid crops, such as cotton, demonstrates that high-density SNP genotyping can substantially enhance QTL detection and enable the development of diagnostic markers for MAS in resistant backcross populations (Venkatesulu et al., 2025), underscoring the need to adopt similar strategies in okra research. Combining MAS for major resistance loci with genome-wide selection (GS) is especially promising for okra improvement, as GS is particularly advantageous for capturing all genetic effects involved in quantitative pest resistance and allows selection based on genomic estimated breeding values each cycle, thereby accelerating genetic gain even in polyploid species with long breeding cycles (Mishra et al., 2017; Singh et al., 2023). However, the application of GS in okra remains limited by the lack of sufficiently large and well-phenotyped training populations. The integration of GS with speed-breeding approaches, such as early generation testing and rapid cycle advancement, has been proposed to further shorten breeding timelines and enhance the accumulation of favorable resistance haplotypes in high-yield, market-preferred genetic backgrounds (Singh and Pandey, 2024). Ultimately, deploying integrated, ploidy-aware MAS and GS systems, supported by expanded genomic resources and detailed phenotyping of jassid damage, will be vital for developing climate-resilient, insect-resistant okra varieties for sustainable agriculture (Mishra et al., 2017; Singh et al., 2023).

The diversity of molecular markers and QTLs associated with jassid resistance in cotton underscores the polygenic architecture of the trait and the involvement of multiple genomic regions (**Table 2**). Both SNP and SSR markers have been reported, reflecting the application of different genotyping platforms. For instance, Venkatesulu et al. (2025) identified SNP markers (i01143Gh, i03879Gh, i02359Gh) on chromosomes 2 and 4 (**Table 2**), indicating that resistance loci are distributed across distinct chromosomal regions. Earlier work by Venkatesulu et al. (2023) detected SSR markers (BL1646 and DOW047), but the absence of chromosomal assignment limits their utility for precise genomic integration. Sankeshwar et al. (2018) mapped two SNP-based QTLs (q-JI-12–1 and q-JI-12-2) on chromosome 12, suggesting the presence of a potentially stable resistance region. Ahmed et al. (2020) reported an expanded set of QTLs linked to stem pubescence (qSPA), leaf pubescence (qLPA), aphid rate (qAR), and aphid grade (qAG), predominantly clustered on chromosome A06, alongside an additional QTL (qAR 1_D09) on chromosome D09. The clustering of resistance-associated loci on A06 suggests a genomic hotspot for insect resistance traits, particularly those mediated by morphological defenses such as pubescence. More recently, Subhashini et al. (2025) identified the SSR marker JESPR154, further validating marker-trait associations for jassid resistance. Overall, these findings indicate that jassid resistance in cotton is controlled by multiple loci distributed across several chromosomes (2, 4, 12, A06, D09). The recurrent detection of QTLs associated with pubescence traits reinforces the pivotal role of morphological mechanisms, notably trichome-mediated defenses, in resistance expression. Importantly, these cotton-derived genomic resources provide a valuable comparative framework for okra, facilitating the identification of orthologous genomic regions, conserved resistance pathways, and candidate genes. Leveraging such cross-species genomic synergies, in combination with advances in polyploid genomics and high-precision phenotyping, holds considerable promise for accelerating the genetic improvement of jassid resistance in okra.

**Table 2:** Summary of molecular markers and quantitative trait loci (QTLs) associated with jassid resistance in cotton.

Species	Insect target	Marker/QTL	Chromosome	References
Cotton	Jassid	SNP: i01143Gh, i03879Gh, i02359Gh	2 and 4	Venkatesulu et al. (2025)
Cotton	Jassid	SSR: BL1646 and DOW047	–	Venkatesulu et al. (2023)
Cotton	Jassid	SNP: q-JI-12-1; q-JI-12-2	12	Sankeshwar et al. (2018)
Cotton	Jassid	SNP: qSPA 1_A06, qSPA 2_A06, qLPA 1_A06, qLPA 2_A06, qLPA 3_A06, qLPA 4_A06, qLPA 5_A06, qAR 1_D09, qAG 1_A06, qAG 2_A06.	A06	Ahmed et al. (2020)
Cotton	Jassid	SSR: JESPR154	–	Subhashini et al. (2025)

### 7.2 Genome-wide association studies

Genome-wide association studies (GWAS) provide a powerful framework for dissecting natural variation underlying jassid resistance in okra at high resolution; however, their effective application requires explicit account for the species’ allopolyploid genome. By exploiting historical recombination in diverse *Abelmoschus esculentus* germplasm, GWAS can identify resistance loci and related defense traits within relatively small linkage disequilibrium blocks and detect multi-allelic variation, thereby overcoming the limited resolution and bi-allelic constraints of biparental QTL mapping (Uffelmann et al., 2021). Robust GWAS in okra will require polyploid-aware statistical models and dense genome-wide SNP datasets generated from high-throughput genotyping platforms, such as genotyping-by-sequencing. These approaches are essential for accurately modeling allele dosage, subgenome structure, and complex LD patterns in okra, while minimizing spurious associations arising from population structure and relatedness (Uffelmann et al., 2021; Gupta et al., 2025). Two decades of GWAS for biotic stress in major crops have demonstrated that resistance to biotic stresses is typically polygenic, involving numerous small-effect loci enriched for genes related to pathogen and insect interactions (NLRs, receptor-like kinases), hormone crosstalk, and downstream defense metabolism (Gangurde et al., 2022). GWAS and multi-omic studies consistently highlight the importance of jasmonic acid (JA) and salicylic acid (SA) signaling components, such as JAZ repressors, MYC transcription factors, MAPK pathways, WRKY, MYB, and AP2/ERF transcription factors, along with genes involved in flavonoid, terpenoid, and glucosinolate biosynthesis, as key regulators of resistance to insects and pathogens (Shrestha and Huang, 2022; Chen et al., 2024). In okra, the development of GWAS panels integrating cultivated and wild *Abelmoschus* species, particularly those characterized for morphological (e.g., trichome density) and biochemical defenses (e.g., phenolics), offers a promising yet underutilized resource for dissecting jassid resistance (Sandhi et al., 2017; Barman et al., 2024). The application of mixed-model GWAS on such panels could enable the identification of robust marker-trait associations that co-localize with candidate genes involved in JA/SA signaling networks. Combining polyploid-aware GWAS with transcriptomics, metabolomics, and gene network analysis will be critical for prioritizing high-confidence candidate genes and validating their functional roles. Such integrative approaches, coupled with improved genomic resources and phenotyping precision, will facilitate the development of tightly linked markers and favorable alleles for marker-assisted and genomic selection, ultimately advancing the breeding of durable jassid-resistant okra varieties.

### 7.3 K-mer-based mapping in okra

K-mer-based mapping has emerged as a promising strategy for dissecting the genetic basis of complex traits in crops with challenging genomic architectures, such as okra. By decomposing raw sequencing reads into short, fixed-length sequences, this alignment-free method captures a wide spectrum of genetic variations, including SNPs, InDels, and large structural variants, without reliance on a high-quality reference genome or prior variant calling (Karikari et al., 2023). This feature is particularly advantageous for okra, a polyploid species with still-developing genomic resources, where conventional SNP-based GWAS approaches are often hindered by paralogous sequence alignment, homeolog interference, and assembly biases (Lemay et al., 2023). Through operating directly on raw sequence data, k-mer-based analysis can circumvent these limitations and improve resolution in structurally complex and repetitive genomic regions. This is especially relevant for resistance loci, nucleotide-binding leucine-rich repeat (NLR) gene clusters, which are frequently associated with pest resistance but are difficult to resolve using standard reference-based approaches (Wiersma et al., 2024; Jaegle et al., 2025).

Despite its considerable potential, k-mer-based mapping remains underutilized in okra, particularly for insect defense, and its application is still in an early stage. To address this gap, we propose a standardized k-mer-based GWAS framework to accelerate the discovery of jassid resistance loci. This approach begins with the assembly of genetically diverse okra panel exhibiting contrasting resistance phenotypes, rigorously validated through multi-environment field trials or controlled infestation assays. Following moderate-depth whole-genome sequencing, k-mers are extracted and filtered to build a presence-absence matrix, which serves as the basis for association analyses that account for hidden relatedness and population structure (Karikari et al., 2023). Significant k-mers are subsequently anchored to available reference genomes or assembled *de novo* from extreme phenotypes to identify candidate loci and resistance-associated gene clusters. However, challenges remain in accurately interpreting k-mer signals and linking them to functional genomic regions. Therefore, downstream validation should integrate multi-omics approaches, including RNA-seq-based expression analysis to confirm candidate genes and elucidate their roles in resistance pathways (Jaegle et al., 2025).

Combining this genomic approach with recent advances in okra phenomics, particularly the use of morphological and biochemical markers of jassid resistance (Barman et al., 2024), creates a powerful synergy. Evidence from related Malvaceae species, such as cotton, highlights the central role of trichome-mediated defenses in insect resistance (Subhashini et al., 2025), suggesting that similar mechanisms may operate in *Abelmoschus*. In this context, k-GWAS has the potential to uncover structural variants and introgressions from wild relatives that underpin these defense traits. Ultimately, shifting to reference-free mapping will facilitate the development of diagnostic markers, improve the efficiency of marker-assisted selection (MAS), and strengthen breeding programs to mitigate evolving jassid pressures, particularly in vulnerable production regions such as West Africa.

### 7.4 Transcriptomics and gene regulatory networks

Transcriptomic and gene regulatory network approaches are increasingly recognized as essential for elucidating the molecular basis of jassid resistance in okra. High-throughput RNA-seq has provided valuable insights into stress-responsive pathways; however, its application in okra remains challenged by polyploidy and gene redundancy (Muthaiah et al., 2024). Evidence from polyploid transcriptomic studies in other species highlights the roles of subgenome expression bias, isoform switching, and selective regulation of duplicated genes under stress conditions (Santoro et al., 2025), yet these dynamics remain insufficiently explored in okra. Under jassid infestation, transcriptomic analyses could be leveraged to distinguish activated and repressed homeologous gene copies, thereby elucidating how polyploidy shapes defense responses. Comparative transcriptomic analyses under controlled jassid infestation and elicitor treatments offer a powerful approach to reconstruct co-expression modules and identify key regulatory hubs, as demonstrated in studies of JA/SA-mediated defense in poplar and Arabidopsis. These studies, combining transcriptome profiling with time-series network modeling, have identified core hormone-responsive genes and key transcription factors in JA signaling pathways (Zhang and Sun, 2021). In particular, components such as JAZ repressors, MYC2-like bHLHs, and other transcription factors, along with genes involved in secondary metabolism, play critical roles in coordinating defense responses through crosstalk with SA and ethylene signaling pathways (Mu et al., 2025; Roychowdhury et al., 2025). In okra, polyploid-aware network inference accounting for duplicated transcription factors and allele-dosage effects will be essential to accurately resolve the organization of JA, SA, and ethylene signaling pathways during jassid attack. This approach could further clarify whether specific homeologs or regulatory modules are preferentially activated in tolerant genotypes. Integrating transcriptomic and network-based analyses will facilitate the identification of mechanistic markers such as inducible defense genes and hormone regulators and provide high-confidence candidate targets for gene editing or expression-based breeding strategies aimed at achieving durable jassid resistance in okra.

## 8 Biotechnological and precision breeding strategies

### 8.1 Genome editing and functional validation

Genome editing, particularly CRISPR/Cas-based systems, represents a powerful extension of precision breeding approaches for improving okra resistance to jassid. However, its effective application requires careful adaptation to the species’ highly complex allopolyploid genome. Evidence from other polyploid crops, including cotton, Brassica, and potato, demonstrates that multiplex CRISPR strategies targeting multiple homeologs can overcome genome redundancy and produce stable phenotypic improvement, including enhanced resistance to insect pests and diseases (Ahmad et al., 2023; Sood et al., 2025). In okra, candidate targets for genome editing can be broadly categorized into two groups: (i) regulatory genes governing defense signaling networks and (ii) structural or biochemical genes underpinning physical and chemical defense mechanisms. Editing key regulatory hubs within JA, SA, and Ca^2+^-mediated signaling pathways has proven particularly effective in enhancing plant resistance. For example, CRISPR/Cas9 mutant libraries targeting calcium-dependent protein kinases (CDPKs) and major latex-like proteins (MLPs) in tetraploid cotton have generated lines with enhanced systemic acquired resistance (SAR) and improved field resistance to multiple insect pests (**Table 3**) (Sun et al., 2024; Wang et al., 2024). The cotton ortholog GhMLP423, in particular, functions as a positive regulator of SA-mediated defense, with overexpression enhancing resistance and gene disruption compromising immunity (**Supplementary Table 1**; Sun et al., 2024). Translating such a strategy to okra through promoter engineering or target gene insertion could reinforce SA-dependent resistance mechanisms against jassids. Similarly, GhCPK33 and GHCPK74 orthologs have been identified as early Ca^2+^-dependent signaling components that regulate JA accumulation and downstream defense activation. CRISPR-based knockout studies in cotton revealed that the disruption of these kinases significantly altered herbivore resistance, highlighting their central role in defense signaling (**Supplementary Table 1**; Wang et al., 2024). Beyond signaling regulators, genes controlling structural defense traits represent key targets for genome editing. Trichome development regulators, including HD-ZIP IV, MYB, and GL2-like transcription factors, are strongly associated with antixenosis-based resistance. Previous studies have consistently shown that genotypes with higher trichome density and length exhibit increased resistance to jassids by reducing feeding and oviposition (**Supplementary Table 1**; Subhashini et al., 2025). Accordingly, CRISPR-based strategies targeting negative regulators or enhancing the activity of positive regulators of trichome formation could improve leaf pubescence and resistance in okra. Biochemical defense pathways also represent promising targets for genome editing intervention. Enzymes involved in the phenylpropanoid/phenolics pathway (e.g., PAL, CHS, 4CL) are directly associated with secondary metabolite accumulation, with elevated phenolic content negatively correlated with jassid and whitefly infestation in okra (**Supplementary Table 1**; Barman et al., 2024). Modulating these pathways through promoter editing or transcriptional activation may therefore enhance both constitutive and inducible chemical defenses. Additional candidate targets include S-adenosylmethionine synthase (SAMS) isoforms, which act as integrative nodes linking methylation, ethylene biosynthesis, and defense signaling. Evidence from cotton indicates that CDPKs interact with SAMS proteins, and that silencing SAMS genes reduces insect defense capacity, suggesting that fine-tuning expression rather than complete knockout would be a more effective editing strategy for optimizing defense responses (**Supplementary Table 1**; Wang et al., 2024). At a broader scale, high-throughput CRISPR screens in cotton targeting insect-responsive differentially expressed genes (DEGs) have revealed numerous loci that either enhance or reduce resistance, further confirming the polygenic nature of insect defense (**Supplementary Table 1**; Sun et al., 2024). This systems-level evidence supports the use of multiplex CRISPR designs in okra to simultaneously enhance positive regulators of defense while eliminating susceptibility factors. Given the allopolyploid nature of the okra genome, achieving complete knockout of susceptibility or structural genes will likely require simultaneous editing of all expressed homeologs, substantially increasing technical complexity. In contrast, promoter or cis-regulatory element modifications of key regulatory genes may offer a more efficient strategy by enabling dosage-sensitive modulation of defense pathways while reducing the number of alleles requiring editing (Zhang et al., 2021; Saeed et al., 2022). However, the successful implementation of such approaches will depend on the development of polyploid-aware genome-editing strategies and efficient validation platforms. Integrating genome editing with rapid functional validation platforms, such as hairy leaves or transient expression systems, will be critical for accelerating candidate gene characterization, as demonstrated in other recalcitrant crops (Alamillo et al., 2023). Comparative insights from cotton provide a valuable framework for identifying orthologous targets and guiding CRISPR/Cas-based interventions to support next-generation okra breeding programs.

**Table 3:** Different secondary metabolites, enzymes, and oxidative responses involved in okra defense against jassid.

Compound/enzymes	Role (antixenosis/antibiosis)	Variation in resistant genotypes	Regulation (constitutive/induced)	Tolerant kra accession	References
Total phenols	Primarily antibiosis (toxicity, harder cell wall) + contribution to antixenosis (less attractive)	Significantly higher levels were observed in hybrids with the fewest jassids.	Primarily constitutive, with an increase after infestation.	Kajari NOH-1684. Japani jhar. Singham. Rohini.	Barman et al. (2024)
Total sugars	Rather, a factor of susceptibility (better food resources)	Higher levels in more susceptible varieties; resistant hybrids have lower total sugar content.	Constitutive	Kajari NOH-1684. Japani jhar. Singham. Rohini (less sugar content than the susceptible).	Barman et al. (2024)
Superoxide dismutase (SOD)	Antioxidant enzyme: a marker of stress rather than a direct cause of resistance (tolerance)	Higher activity in heavily infested varieties; tolerant genotypes show lower SOD activity	Induced	Kajari NOH 1684. Japani jhar. Singham. Rohini (lower SOD, despite lower infestation).	Barman et al. (2024)
Peroxydase (POD)	ROS detoxification, possible tissue hardening (antibiosis)	Significantly higher activity in heavily infested varieties; tolerant varieties exhibit lower POD activity	Induced	Same panel of hybrids with low activity in Kajari NOH 1684. Japani jhar. Singham. Rohini.	Barman et al. (2024)
Tanins	Primarily antibiosis (toxicity, harder cell wall) + contribution to antixenosis (less attractive)	Higher in resistant varieties	Primarily constitutive, with an increase after infestation.	EC0306728.	Sandhi et al. (2017)

### 8.2 Speed breeding and phenomics integration

Speed breeding and phenomics integration represent a logical progression in biotechnological and precision breeding pipelines for developing jassid-resistant okra, particularly in light of its long breeding cycles and complex polyploidy genome. Manipulation of environmental conditions, such as photoperiod, temperature, and light intensity, can shorten generation time, enabling up to 4–6 generations per year in several cereals and oilseed crops, thereby dramatically accelerating annual genetic gain and trait introgression (Song et al., 2021; Samantara et al., 2022). Similar rapid-cycling protocols have been combined with multi-trait phenotyping platforms for disease resistance and root architecture in durum wheat, demonstrating that effective selection for stress-related traits is feasible under speed-breeding conditions (Alahmad et al., 2018). Stochastic simulations in wheat further indicate that integrating speed breeding with genomic selection can significantly enhance the response to selection for complex quantitative resistance, as illustrated for Fusarium head blight resistance (Nannuru et al., 2025). Critically, speed breeding platforms increasingly incorporate high-throughput, often image-based phenotyping systems to capture growth, morphology, and stress responses at scale, thereby addressing the longstanding phenotyping bottleneck in insect-resistance breeding (Bhat et al., 2023; Patel and Gurjar, 2024). Camera-based and miniaturized phenotyping assays for insect damage assessment, such as those used to assess pollen beetle resistance in oilseed rape, illustrate how standardized mid- to high-throughput screening systems can generate robust and quantitative resistance phenotypes for QTL mapping and selection, even in polyploid crops (Obermeier et al., 2022). For okra, adapting controlled-environment, speed-breeding systems integrated with high-throughput phenomics offers a promising yet underdeveloped avenue to accelerate resistance breeding. The use of automated imaging platforms to quantify jassid feeding damage, leaf pubescence, vigor recovery, and other defense-related traits across rapid growth cycles would allow efficient recycling of segregating generations while maintaining sufficient population sizes to capture the complexity of its polyploid genome. When coupled with MAS or GS, these integrated pipelines have the potential to substantially shorten breeding cycles, improve the precision of resistance selection, and accelerate the development of stable, jassid-resistant okra cultivars suited for sustainable production systems.

## 9 Integrated pipeline for jassid-resistant okra breeding

**Figure 7:**
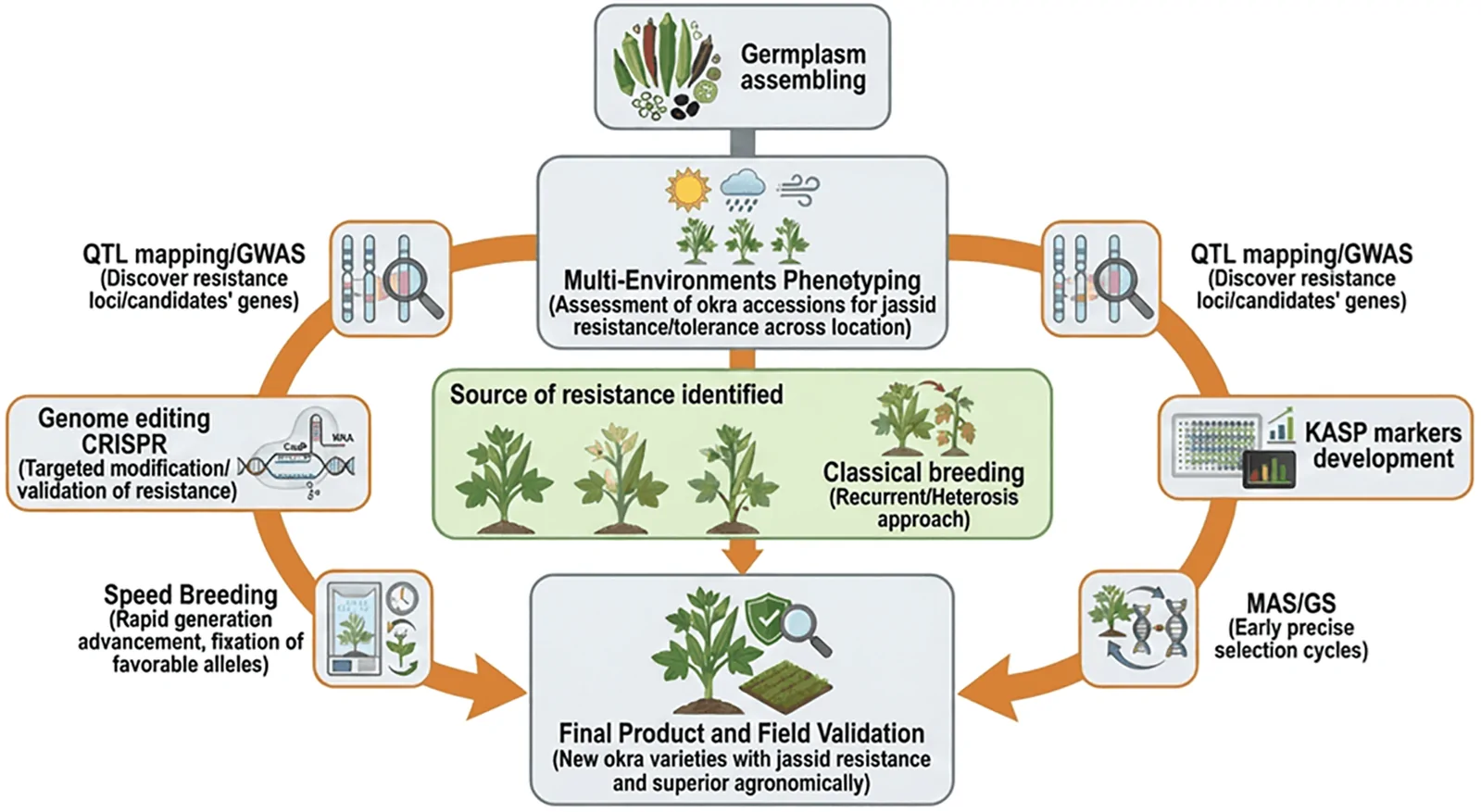
Integrated breeding pipeline for resistance to jassids.

The integrated breeding pipeline for jassid-resistant okra is a systems-oriented, multi-step framework that strategically combines conventional breeding with advanced genomic tools, genome editing, and accelerated breeding approaches to deliver durable resistance. The process begins with rigorous multi-environment phenotyping, in which diverse okra accessions are evaluated across locations and seasons to identify genotypes with stable resistance while reducing confounding genotypes by environment interactions, thereby establishing a robust phenotypic foundation for downstream genomic analyses (**Figure 7**). These phenotypic data are subsequently integrated into quantitative genetic analyses, including QTL mapping and GWAS, to identify resistance-associated loci and candidate genes (**Figure 7**). The resulting loci can then be deployed through MAS and genomic selection (GS), enabling precise, early-generation identification of superior lines and shortening the breeding cycle. In parallel, CRISPR-based genome editing offers a complementary strategy for targeted modification of key resistance genes (**Figure 7**). Selected elite lines are then advanced under speed-breeding conditions to accelerate generation turnover and facilitate the fixation of favorable alleles. Final validation under natural jassid infestation remains essential to confirm both the stability of resistance and agronomic performance (**Figure 7**). In addition, validated resistant sources are introgressed into conventional breeding schemes, such as recurrent selection and heterosis-based hybrid development, ensuring the practical deployment of resistance traits. Ultimately, the success of this pipeline depends on the effective integration of genomic tools with classical breeding approaches, supported by robust phenotyping and functional validation. Strengthening each component of this framework will be critical for delivering resilient, farmer-ready okra cultivars with durable jassid resistance (**Figure 7**).

## 10 Designing jassid-resistant okra ideotypes

Designing ideotypes for jassid-resistant okra requires pyramiding complementary traits across morphological, biochemical, and genetic dimensions to ensure that multiple resistance mechanisms act synergistically without compromising yield and pod quality. Morphological traits such as high midrib hair density, long and erect trichomes, broad leaves, and optimized leaf architecture are consistently associated with reduced jassid infestation in *Abelmoschus* species, indicating a strong antixenosis-based resistance ideotype (Sandhi et al., 2017; Acharya et al., 2019). Biochemically, elevated phenols, tannins, silica, and balanced sugar profiles contribute to antibiosis and reduced jassid build-up. Similar multi-trait “physico-chemical” resistance complexes have already been exploited in okra lines tolerant to whitefly and fruit and shoot borer (Acharya et al., 2019; Barman et al., 2024). At the genetic level, these morphological and biochemical traits can be reinforced by pyramiding resistance genes and QTLs from wild *Abelmoschus* species and tolerant accessions (e.g., *A. tetraphyllus*, *A. angulosus*, *A. moschatus*, BCO-1, Arka Anamika). This strategy aligns with gene-pyramiding approaches widely used to enhance the durability of insect resistance in cotton, maize and other crops (Sandhi et al., 2017; Dormatey et al., 2020; Razzaq et al., 2023). However, ideotype design must explicitly balance resistance with other traits in the product profiles for diverse market segments. Multivariate and diversity studies in okra show that high yield, seed protein, fiber, and other nutritional traits can be improved concurrently, indicating that resistance can be effectively combined with value-added traits rather than requiring trade-offs (Mohammed et al., 2022; Jyothsna et al., 2024; Ayenan et al., 2025). Importantly, the success of any ideotype ultimately depends on its acceptance by farmers and consumers. Participatory assessments in okra-growing regions consistently highlight farmers’ preferences for high yield, pest resistance, marketability, and drought tolerance, while consumers increasingly prioritize tender pods with good flavor, low fiber content, and enhanced nutritional value (Ibitoye and Kolawole, 2022; Ayenan et al., 2025). Thus, the added value of a jassid-resistant okra ideotype lies in integrating multi-layered resistance with superior pod quality, nutritional attributes, and agronomic performance. Co-designing such ideotypes with stakeholders will be critical to ensure their relevance, adoption, and impact, particularly within smallholder production systems.

**Figure 8:**
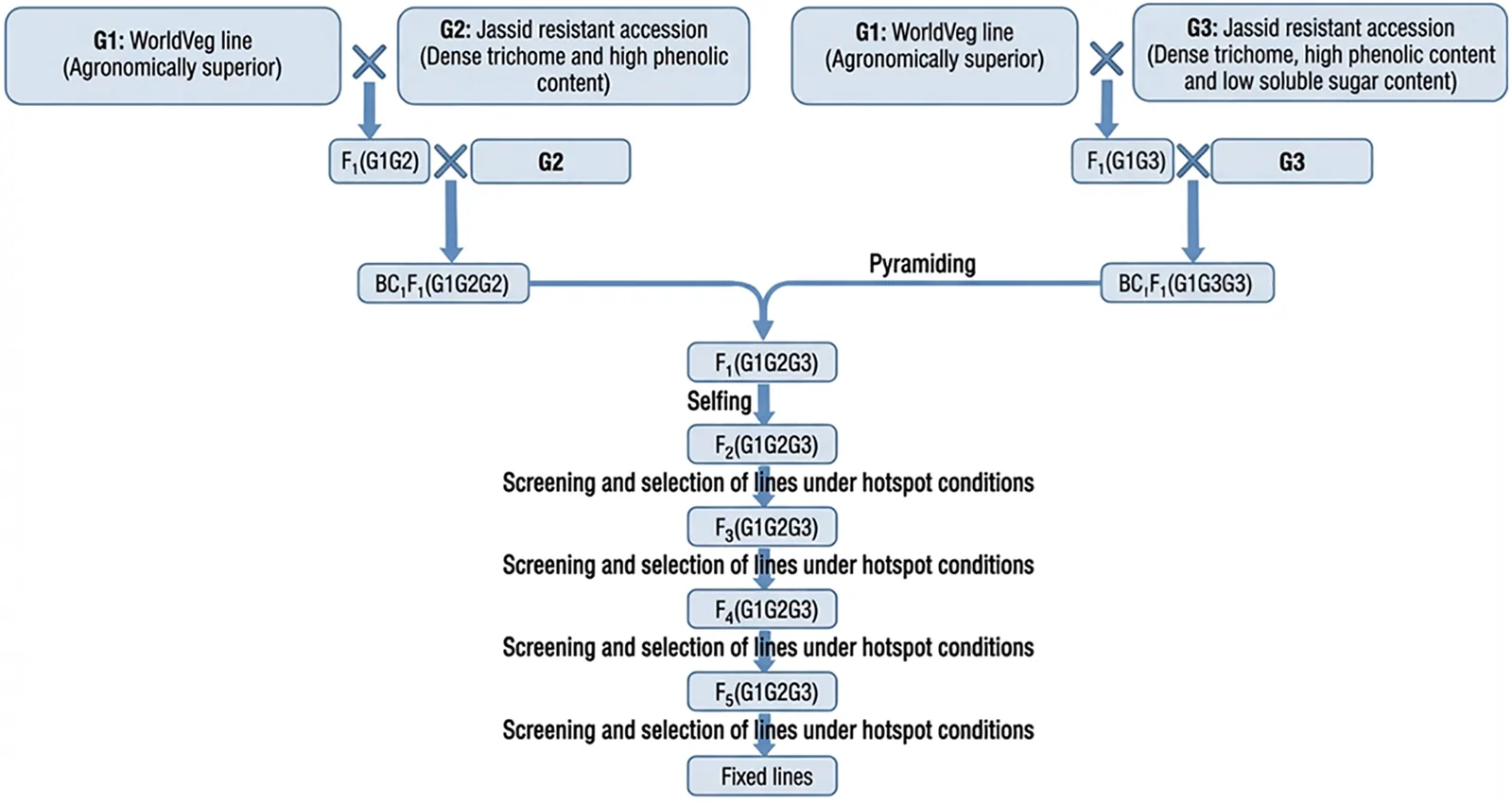
Okra breeding pipeline for developing jassid-resistant lines.

A stepwise breeding strategy has therefore been proposed; it involves crossing an agronomically superior cultivated okra line (G1; WorldVeg) with two resistant donor lines (G2 and G3) characterized by dense trichomes and high phenolic content associated with jassid resistance (**Figure 7**). Initial crosses (G1 × G2 and G1 × G3) produce F_1_ hybrids, which are subsequently backcrossed to the elite recurrent parent to recover desirable agronomic traits while introgressing resistance traits, generating BC_1_F_1_ populations (**Figure 8**). These lines are subsequently intercrossed to pyramid complementary resistance alleles, followed by accelerated selfing and selection from F_2_ to F_5_ in hotspot environments. Selection targets resistance-associated morphological and biochemical traits, including dense erect trichomes, thick stems, pubescent leaves, elevated phenolic content, and reduced soluble sugars, alongside agronomic traits such as compact architecture, early flowering, and high fruit set. This pipeline aims to develop an optimized okra ideotype combining durable jassid resistance with high yield potential and acceptable horticultural quality (**Figure 8**).

We also propose a multi-cycle recurrent selection scheme utilizing an elite founder pool characterized by quantitative, moderate resistance to jassid. This strategy facilitates the progressive recombination of minor-effect quantitative trait loci (QTLs), effectively increasing the frequency of favorable alleles. This approach optimizes the selection differential for durable, polygenic resistance without compromising the genetic gain for yield-component traits.

## 11 Challenges and future directions for jassid resistance in okra

Designing durable jassid-resistant okra faces intertwined challenges in germplasm characterization, phenotyping, genomics and translation to farmers’ fields. Although more than 5340 okra germplasm accessions exist worldwide (Ayenan et al., 2025), they remain insufficiently and unsystematically characterized. A comprehensive evaluation to develop core collections is essential to streamline research efforts and accelerate the identification of resistant genotypes. Phenotyping for jassid resistance is intrinsically complex because pest pressure, plant vigor and physico-chemical traits (trichomes, phenols, sugars) all vary with season, microclimate, and plant stage, making resistance classifications unstable across years and locations (Acharya et al., 2019; Barman et al., 2024). Similar difficulties in reliably scoring insect damage and plant responses have slowed resistance breeding in oilseed rape and vegetables, where insect performance and damage traits are laborious, low throughput and often poorly linked to field outcomes (Brzozowski and Mazourek, 2020; Obermeier et al., 2022). For okra, these phenotyping constraints are compounded by limited genomic resources and reference panels: the crop’s complex polyploid genome, sparse markers, and absence of robust linkage maps or large, well-phenotyped diversity panels restrict QTL mapping and genomic selection for insect resistance (Mishra et al., 2017; Singh and Pandey, 2024; Ayenan et al., 2025). Although transcriptomic resources and initial GWAS in okra are emerging, they have not yet been systematically linked to resistance phenotypes or deployed in breeding pipelines (Schafleitner et al., 2013; Sun et al., 2023; Xu et al., 2024; Schafleitner and Lin, 2025).

Future direction to discover QTLs associated with jassid resistance will require multi-location, multi-omics integration that explicitly captures genotype × environment × pest interactions. Experience in rice, legumes and climate-resilient crops shows that combining genomics, transcriptomics, metabolomics, and high-throughput phenomics with environmental covariates and machine-learning models can greatly improve prediction of complex resistance traits and dissect G×E effects (Gothe et al., 2024; Ontoy and Ham, 2024; Amin et al., 2025; Creach et al., 2025; Mohamedikbal et al., 2025). Comparable frameworks are currently lacking for okra, where even basic pest hotspot mapping, disease/pest diversity characterization, and standardized digital phenotyping protocols remain underdeveloped (Ayenan et al., 2025). Addressing these gaps will benefit from coordinated, cross-institutional networks, including international jassid phenotyping nurseries, harmonized scoring protocols, and open, genomic and phenotypic databases dedicated to okra. Finally, there is a major translational gap from lab to field: omics-based markers, candidate genes, and even genome-editing concepts for insect resistance are advancing rapidly in major field crops, but few have been validated under farmer conditions or integrated into low-input, smallholder-focused okra breeding schemes (Tyagi et al., 2020). Bridging these gaps will require not only investments in okra-specific genomic infrastructure and standardized multi-environment phenotyping for jassid resistance, but also collaborative breeding platforms and participatory trials that link global discovery efforts to locally adapted, resilient cultivars that can be widely adopted.

## 12 Conclusion

Jassids remain among the most destructive pests constraining okra production, and their impact is intensifying in tropical and subtropical regions as climate change is projected to expand their range and increase population pressures. Synthetic insecticides currently dominate control efforts, but over-reliance could drive resistance development, environmental degradation, rising production costs, and health risks, underscoring the need for durable host-plant resistance as a central pillar of sustainable management. As synthesized in this review, resistance is governed by morphological, biochemical, and physiological mechanisms conferring antixenosis and antibiosis. Cultivated and wild *Abelmoschus* species harbor a wealth of resistance alleles that remain critically underutilized. Advances in genomics and precision breeding tools offer unprecedented opportunities to accelerate cultivar development. Nevertheless, key gaps persist: the genetic architecture of resistance remains poorly resolved, validated QTLs and functional genes are scarce, and okra’s genomic infrastructure lags behind that of other major vegetable crops. Future research should prioritize (i) discovery and functional validation of resistance genes; (ii) systematic exploration and introgression of wild Abelmoschus germplasm; (iii) integration of multi-omics with high-throughput phenotyping; and (iv) multi-environment trials across diverse jassid biotypes to secure broad-spectrum, durable resistance. Developing ideotypes that pyramid multiple resistance mechanisms while sustaining agronomic performance, and deploying them within climate-smart production systems, offers the most viable path to reducing pesticide dependence, conserving biodiversity, enhancing resilience to climate volatility, and securing smallholder livelihoods across the tropics.
