# Supplementary material for: Breeding jassid-resistant okra (Abelmoschus esculentus): from morpho-biochemical traits to precision breeding
Source: Front Plant Sci. 2026 Aug 13;17:1897976. doi: 10.3389/fpls.2026.1897976 (PMC13520211; doi:10.3389/fpls.2026.1897976)
Supplement: Supplementary file 1 [file Table1.docx]

Supplementary Material

**Supplementary Table 1.** Potential target genes identified in cotton that may serve as candidates in okra based on orthologous relationships.

| **Putative gene class in okra (ortholog source)** | **Expected role in resistance to jassids** | **Suggested CRISPR strategy in okra (gain/loss of function)** | **Rationale from cotton / other species** | **Reference** |
| --- | --- | --- | --- | --- |
| Trichome-development regulators (e.g. HD-ZIP IV, MYB, GL2-like; inferred from cotton hairy vs glabrous genotypes) | Increased leaf hair density/length to deter feeding and oviposition (antixenosis) | Knock-out repressors of trichome formation or activate positive regulators using CRISPR activation/base editing | Cotton jassid-resistant lines show higher trichome density and length; pubescent genotypes with JESPR154-tagged locus are more resistant to jassids | Subhashini et al. (2025) |
| Phenylpropanoid / phenolics pathway enzymes (PAL, CHS, 4CL, etc.) | Higher constitutive/induced phenolics reduce jassid/whitefly population and damage | Up-regulate key biosynthetic genes (promoter editing) or knock out negative regulators | In okra, higher total phenols are negatively correlated with jassid and whitefly infestation; resistant varieties show elevated phenols | Barman et al. (2024) |
| Major latex-like protein 423 ortholog (GhMLP423 → AeMLP-like) | Positive regulator of broad-spectrum insect resistance via SA-dependent systemic acquired resistance (SAR) and ROS/Ca²⁺ waves | Create overexpression alleles (promoter editing, knock-in) or remove inhibitory cis-elements | Overexpression of GhMLP423 in cotton enhances resistance to multiple insect pests by activating SA-dependent SAR and PR genes; knockouts reduce resistance | Sun et al. (2024) |
| CDPKs involved in herbivory signaling (GhCPK33/74 → AeCPK33/74-like) | Early Ca²⁺-signaling nodes triggering jasmonic-acid and downstream defense against feeding | Generate gain-of-function or hyperactive alleles of positive CDPKs; avoid disrupting beneficial members | In cotton, CRISPR knockout library of GhCPKs identified cpk33/74 mutants with strongly altered chewing-insect resistance; oral secretions induce Ca²⁺ influx and JA accumulation via these kinases | Wang et al. (2024) |
| S-adenosylmethionine synthase (SAMS) isoforms (GhSAMS1/2 → AeSAMS1/2-like) | Central nodes for methylation and ethylene/defense signaling impacting herbivore resistance | Fine-tune expression (promoter editing) rather than full loss-of-function | Cotton GhCPK33/74 interact with GhSAMS1/2; silencing SAMS genes compromises defense against Spodoptera, indicating a key role in insect resistance signaling | Wang et al. (2024) |
| General insect-induced defense genes from cotton DEG sets (502-gene CRISPR library) | Diverse receptors, signaling components, and defense proteins responding to sap-sucking insects | Prioritized multiplex editing to enhance known positive regulators or remove susceptibility factors | A high-throughput cotton CRISPR library targeting 502 insect-responsive genes identified numerous mutants with decreased or increased resistance, including broad-spectrum loci | Sun et al. (2024) |
| Pathogenesis-related (PR) proteins (PR1, PR2, PR5 orthologs) under SA control | Strengthen basal and systemic resistance to sap-sucking insects | Promoter engineering for stronger inducible expression | GhMLP423-mediated SAR in cotton up-regulates SA-dependent PR genes, correlating with enhanced insect resistance | Sun et al. (2024) |
| Redox enzymes (peroxidases, SOD regulators) | Modulate ROS bursts and oxidative signaling during insect attack | Edit regulatory regions to optimize timing/magnitude of ROS | In okra, higher SOD and peroxidase activities are associated with pest attack; modulation likely part of tolerance vs susceptibility | Barman et al. (2024) |
